# Research Trends and Emerging Frontiers in Racehorse Genetics: A Bibliometric Analysis

**DOI:** 10.3390/ani16111705

**Published:** 2026-06-02

**Authors:** Qiuping Huang, Xinkui Yao, Xue Yang, Tianwei Wang, Xinlong Li, Dehaxi Shan, Yi Su, Jianwen Wang

**Affiliations:** 1 College of Animal Science, Xinjiang Agricultural University, Urumqi 830052, China; 2 Xinjiang Key Laboratory of Equine Breeding and Exercise Physiology, Urumqi 830052, China; 3 Sichuan Animal Science Academy, Chengdu 610066, China; 4 Animal Genetic Breeding and Reproduction Key Laboratory of Sichuan Province, Chengdu 610066, China

**Keywords:** horse racing, equine genetics, genetic diversity, bibliometric analysis, hot trends

## Abstract

The global horse racing industry relies heavily on elite breeding. Therefore, a molecular understanding of athletic traits is crucial for the industry’s advancement. This study utilized Bibliometrix, VOSviewer, and CiteSpace to conduct a comprehensive bibliometric analysis of 869 publications in the Web of Science Core Collection (2011–2025), decoding the academic landscape and evolutionary trajectory of equine genetics. The results indicate a significant upward trend in academic output since 2019, with the United States, South Korea, and the United Kingdom emerging as the primary contributors. Institutions such as the University of California, Davis, and University College Dublin serve as key hubs within a highly structured global collaborative network. Analysis of keyword co-occurrence and bursts reveals a distinct paradigm shift: research has evolved from early single-gene identification (e.g., MSTN) to large-scale genome-wide scans, and more recently to functional genomics and bioethical issues. Contemporary research frontiers increasingly focus on “gene doping” “homozygosity” and “risk factors” signaling that horse genetics is shifting from performance-oriented selection toward a comprehensive framework that integrates phenotypes, health, and genetic diversity. This study provides researchers with a strategic roadmap to help them align with emerging trends and foster international collaboration in equine genomic selection.

## Simple Summary

Racehorse genetics has become an important research area for improving athletic performance and maintaining genetic diversity. However, the global development patterns and emerging research frontiers remain unclear. This study applied bibliometric methods to analyze racehorse genetic research from 2011 to 2025. The results revealed a rapid growth in publications, strong international collaboration networks, and a clear shift from single-gene studies to genome-wide and multi-omics approaches. Emerging topics such as gene doping, homozygosity, and risk factors indicate increasing attention to genetic safety and sustainable breeding. These findings provide insights for future research and precision breeding strategies in racehorses.

## 1. Introduction

As a vital component of the global livestock economy, the horse racing industry is a distinctive sector that integrates competitive sports, cultural heritage, and economic value. Breeding for superior traits is the cornerstone for enhancing horses’ competitive performance, preserving high-quality genetic resources, and driving the industry’s high-quality development [1,2]. In traditional breeding models, selection primarily relies on phenotypic observation, breeding experience, and progeny testing. These methods are time-consuming and limited in precision, making it difficult to efficiently decipher the underlying genetic mechanisms of core competitive traits such as speed, endurance, muscle development, and exercise tolerance [3,4]. With the rapid advancement of molecular genetics and genomics technologies, the three core theoretical and technical frameworks—genomics, gene expression, and heritability—have become the key pillars for unraveling the genetic mechanisms underlying superior traits in racehorses and achieving precision breeding [5]. Genes are the fundamental functional units that carry the genetic information of racehorses and determine germplasm characteristics; they directly govern the inherent genetic potential of horses in terms of growth and development, muscle differentiation, and energy metabolism [6,7]. Heritability, as a quantitative indicator, can precisely determine the proportion of key competitive traits—such as speed and endurance—that are genetically regulated. It clarifies the potential for stable improvement and intergenerational transmission of these traits through directed selection, serving as a crucial basis for formulating mating plans, estimating breeding values, and selecting core sires [8,9]. Gene expression, meanwhile, bridges genetic information with actual phenotypes. Through the spatiotemporal regulation of transcription and translation, it elucidates how genes ultimately influence a horse’s athletic performance, physical reserves, and environmental adaptability, thereby revealing the molecular regulatory mechanisms underlying the formation of superior traits [7,10]. Genomics, gene expression, and heritability provide a solid foundation for precision breeding in racehorses. The identification of performance-related genes, the elucidation of gene expression regulatory mechanisms, and the precise estimation of trait heritability will greatly promote the healthy development of the racehorse industry [5].

Bibliometric analysis has also been applied to the broader field of animal breeding [11], revealing trends, key contributors, research hotspots, and patterns of interdisciplinary integration within specific research areas [12]. As a key branch at the intersection of life sciences and animal science, the field of horse breeding genetics warrants a systematic review and summary of its academic output and development trends through bibliometric methods [13]. Based on the Web of Science Core Collection (WoSCC), this study comprehensively employs bibliometric tools such as Bibliometrix, VOSviewer, and CiteSpace to conduct an in-depth analysis of global research outcomes in the field of horse genetics from 2011 to 2025. The aim of this paper is to systematically trace the logical pathway of the field’s transition from “basic genetic discoveries” to “complex regulatory networks” and to identify current research hotspots and emerging trends.

## 2. Materials and Methods

### 2.1. Database Selection and Advanced Search Strategies

To ensure the comprehensiveness and academic authority of the research dataset, data for this study were retrieved from the Science Citation Index Expanded (SCIE) within the Web of Science Core Collection (WoSCC) [14]. The search strategy employed a Boolean combination of titles, abstracts, and keywords (TS) to capture the multidimensional nature of horse racing genetics. The search query was constructed as follows: TS = (“racehorse*” OR “race horse*” OR “horse racing” OR “Thoroughbred*” OR “Standardbred*” OR “equine athlete*”) AND TS = (“gene” OR “genes” OR “genetic*” OR “genomic*” OR “DNA” OR ‘transcriptome’ OR “genome-wide” OR “GWAS” OR ‘heritability’ OR “polymorphism”). The time span was set from 1 January 2011 to 31 December 2025, with the aim of comprehensively documenting the development of equine science during the “genomic era”. To reduce “noise” in the dataset, the document types were strictly limited to original research articles and reviews, excluding online-only publications, conference proceedings, book chapters, and retracted publications.

### 2.2. Data Refinement and Standardization of Intellectual Property

To address the inherent challenges of “synonymy” and “polysemy” in bibliometric metadata, this study implemented a rigorous three-step data cleaning protocol. To resolve author disambiguation, a custom thesaurus file was constructed to merge different name entries for the same author (e.g., unifying “Hill, E.”, “Hill, Emmeline”, and “Hill, EW”). Author identities were cross-verified using ORCID identifiers and institutional records to ensure the rigor of h-index and citation metrics. During institutional consolidation, variants of institutional names (e.g., “Univ Calif Davis” and ‘UCDavis’) were normalized and merged into their parent organizations to accurately reflect regional research strength. In keyword standardization, semantically similar terms (e.g., “messenger RNA” and “mRNA”) were merged to prevent dilution of keyword emergence strength.

### 2.3. Synergies Between the Analytical Framework and Software

This study employs methodological triangulation [15] to integrate the algorithmic strengths of three specialized platforms, aiming to achieve comprehensive coverage and in-depth analysis of the research perspective. Using Bibliometrix (R package, v.4.5.3) for macro-level quantitative mapping [16], we calculated annual literature growth rates, verified the fit between author productivity and Lotka’s Law, and utilized the Three-Field Plot to reveal knowledge flow pathways among countries, institutions, and subject areas. Regarding the construction of relational networks, VOSviewer (v.1.6.20) [17] was employed to perform distance-based clustering analysis. Through VOS mapping technology, the spatial distance between nodes directly reflects academic proximity. Unlike traditional topological structures, this approach uses Total Link Strength (TLS) as the core metric for measuring collaborative networks. Concurrently, CiteSpace (v.6.2.R2) was employed to conduct dynamic frontier detection [18], combining the Kleinberg emergence detection algorithm to identify emerging research trends. The Timeline View was utilized to track the evolution of the knowledge base, thereby precisely locating academic bridge nodes with high “betweenness centrality” that connect different.

## 3. Results

### 3.1. Overview of Overall Output in Thoroughbred Horse Genetics Research

#### 3.1.1. Analysis of Annual Publication Volume Trends

This study included a total of 869 literature sources on thoroughbred horse genetics research. In terms of publication type distribution, the data comprised 819 original research articles and 50 review articles, accounting for 94.25% and 5.75% of the total, respectively. A total of 3691 authors were involved, with international collaboration accounting for 29.46% of the publications. Among these articles, 98.27% (854 articles) were published in English, 8 in German, 3 in French, 2 in Italian, and one each in Portuguese and Italian.

**Figure 1:**
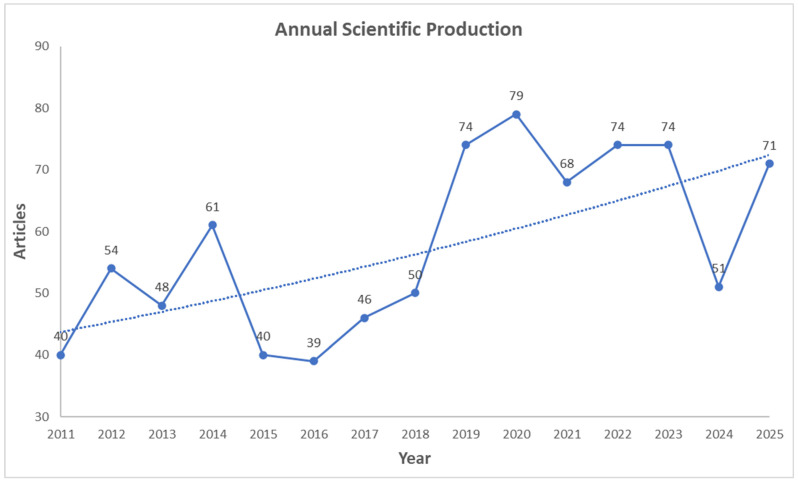
Annual publication trends in the field of horse genetics from 2011 to 2025. The solid line and data points represent the specific number of publications each year, while the dashed line illustrates the overall upward trend in this field over the past 15 years.

As shown in Figure 1, the annual publication volume of horse genetics research from 2011 to 2025 was compiled. The results indicate that academic output in this field has shown a steady upward trend overall. The period from 2011 to 2018 represented an initial growth phase, with the annual average publication volume remaining below 50. Starting in 2019 (74 articles), research output entered a phase of rapid growth, peaking in 2020 (79 articles). Subsequently, from 2022 to 2025, the annual publication volume remained stable at a high level of over 70 articles, indicating that horse genetics and genomics are currently in an active phase of development and continue to attract significant attention from the academic community.

#### 3.1.2. Changes in Publication Output by Country over Time and Maps of Regional Distribution

**Figure 2:**
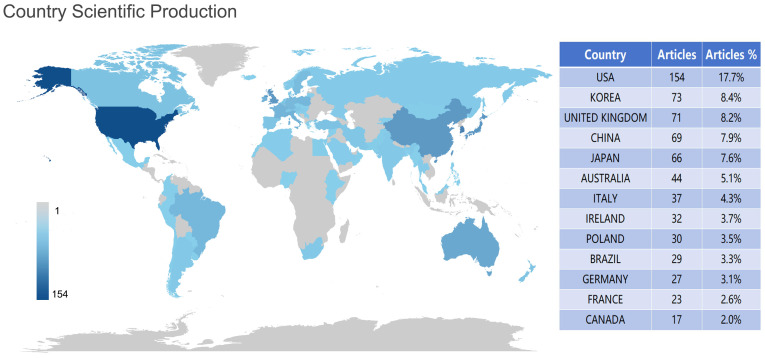
Country Scientific Production. A world map illustrating the contributions of various countries based on the number of research papers published. The color gradient on the world map represents the volume of publication output (number of articles), ranging from dark blue (highest production) to light blue (lowest production); grey areas indicate no data available.

An analysis of the geographical distribution and temporal evolution of publications reveals that horse racing genetics research is a highly globalized field. The United States, South Korea, and the United Kingdom are the core contributors to this field, and their publication output has consistently led the field over the past 15 years. As shown in Figure 2, the map of country and regional distribution and the table of publication statistics indicate that research in this field exhibits a globalized character spanning multiple continents. Among these, the United States (USA) holds an absolute, dominant position with a total of 154 publications, followed closely by South Korea (73), the United Kingdom (UK, 71), China (69), and Japan (66), which rank among the top five globally in terms of publication volume. The map of the country and regional distribution clearly illustrates that North America, East Asia, and Western Europe constitute the three major core clusters of global horse genetics research.

### 3.2. Analysis of Core Research Capabilities and Collaborative Networks

#### 3.2.1. Analysis of Core Journal Influence

**Figure 3:**
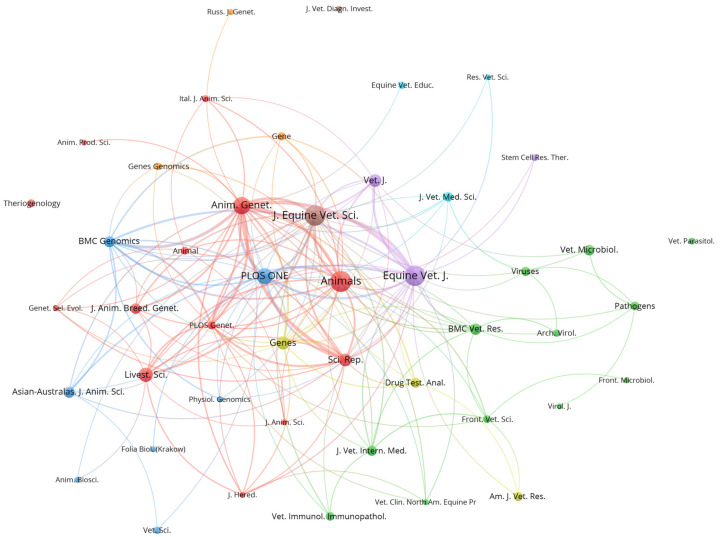
A bibliometric network diagram of citation sources in horse genetics research. Each node represents a journal, and the size of each node reflects the journal’s total link strength or citation frequency within the dataset. The lines connecting the nodes indicate citation relationships. The colors, based on a clustering algorithm that groups journals into different thematic clusters, illustrate the interdisciplinary landscape spanning veterinary medicine, animal science, and genomics.

The analysis of journal distribution and co-citation networks (Table 1, Figure 3) reveals a highly integrated knowledge structure in this field. *Animals* (57 articles), *Equine Veterinary Journal* (54 articles), and *Animal Genetics* (37 articles) are the most influential core journals in this field. Research on horse genetics exhibits distinct clustering characteristics and strong inter-journal connections. Core journals such as *Animals*, *Equine Veterinary Journal*, and *PLOS ONE* are located at the center of the network, with larger nodes and tight connections, indicating their significant influence and foundational role in the field. The differently colored clusters primarily cover research directions such as equine veterinary medicine, animal genetics and genomics, molecular biology, and veterinary microbiology. The close connections between these clusters reflect a distinct trend toward interdisciplinary convergence in horse genetics research, with a focus on areas such as genomic analysis, regulation of athletic performance, disease mechanisms, and molecular regulatory networks.

#### 3.2.2. Output and Collaboration Characteristics of Core Authors

**Figure 4:**
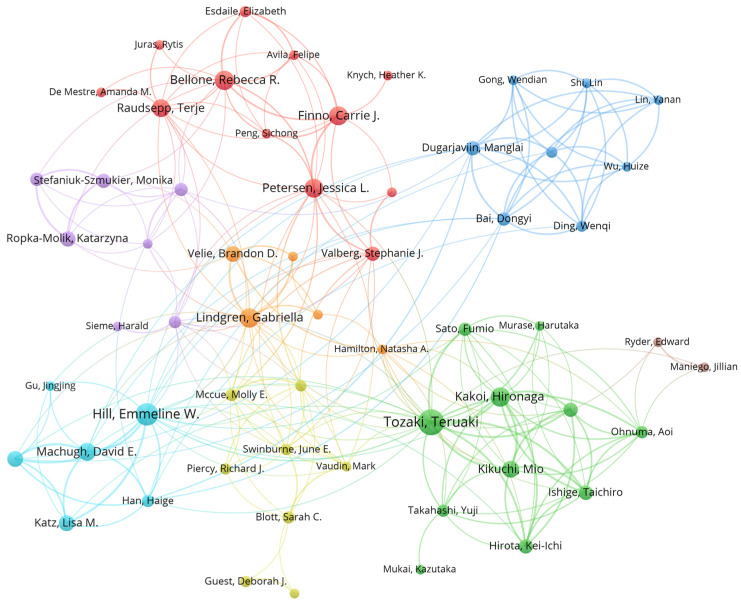
A visualization of the co-authorship network among influential researchers in the field of equine genetics. Node size represents the number of publications by each author, line thickness indicates the strength of collaboration between authors, and different colors denote distinct collaboration clusters, revealing core research teams and their collaborative relationships.

An in-depth analysis of core authors and their collaborative networks (Table 2, Figure 4) highlights the existence of a robust academic community in the field of horse genetics research. Emmeline W. Hill tops the list with 32 publications, closely followed by Teruaki Tozaki with 31 publications, further solidifying their status as the most influential and authoritative experts in the field. VOSviewer network visualization further reveals several distinct disciplinary clusters, indicating that although global research efforts are highly specialized, they remain closely interconnected. Specifically, the light blue cluster centered on Hill, EW primarily explores exercise physiology and performance genetics, while the green cluster led by Tozaki, T focuses on equine genomics and population genetics, reflecting the active contributions of East Asian research institutions. Furthermore, the thick links observed between core nodes—such as Hill, Tozaki, and Petersen—highlight significant knowledge flow across national and disciplinary boundaries, which continues to drive the discovery of molecular mechanisms underlying key traits in racehorses.

#### 3.2.3. Distribution of Key Institutions and Global Collaboration

**Figure 5:**
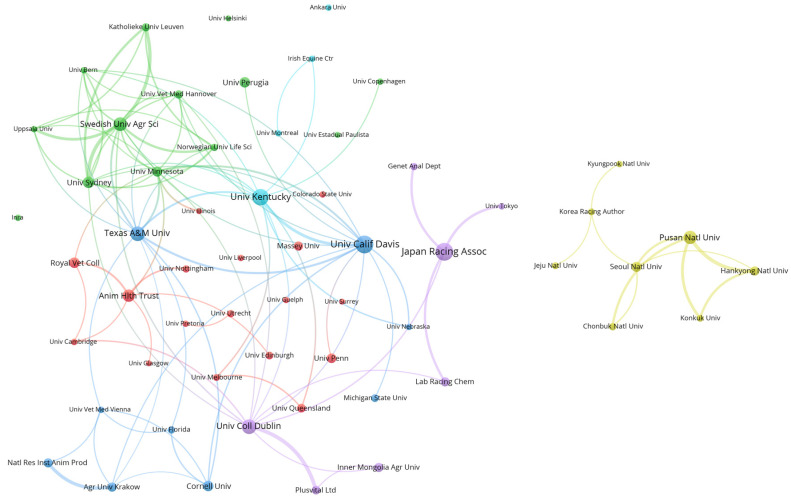
Visualization of the collaborative network among institutions in the field of horse genetics. Node size indicates the number of publications by each institution, line thickness represents the strength of collaboration between institutions, and different colors denote different collaborative clusters, reflecting the collaborative relationships among major research institutions in this field and the landscape of international collaboration.

Combining publication statistics (Table 3) with an analysis of institutional collaboration networks (Figure 5) reveals that research on horse genetics has evolved into a global landscape dominated by European and American institutions, with Asian institutions developing rapidly. The University of California System and the University of California, Davis rank at the top with 111 and 103 publications, respectively, and occupy central hub positions in the collaboration network. They maintain close collaborations with institutions such as the University of Kentucky, Texas A&M University, and University College Dublin, demonstrating strong academic influence and resource integration capabilities. In Europe, institutions such as the Swedish University of Agricultural Sciences have formed a stable collaborative group; in Asia, represented by Pusan National University and Seoul National University, a relatively independent yet closely connected regional collaborative network has been established. Overall, the connections between institutions are strong, and cross-regional collaboration has significantly increased, indicating that international cooperation has become a key driver for the continuous deepening and innovative development of horse genetics research.

### 3.3. Thematic Evolution and Frontier Exploration

#### 3.3.1. Keyword Evolution and Co-Occurrence Analysis

**Figure 6:**
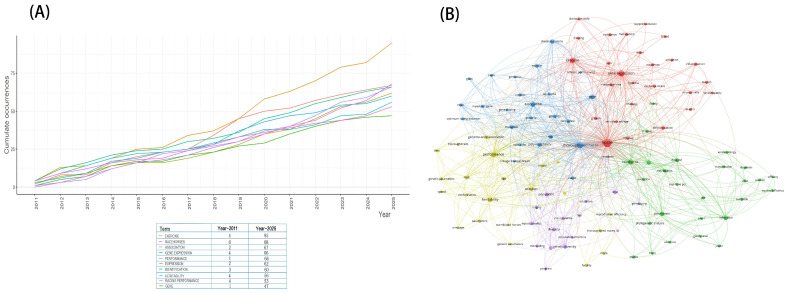
Dynamic Evolution and Co-occurrence Network of Keywords in Horse Racing Genetics Research (2011–2025) (**A**) The line chart shows the cumulative frequency of the top 10 high-frequency keywords over a 15-year period. (**B**) Visualization of the keyword co-occurrence network. Each node in the figure represents a keyword, with node size reflecting its frequency of occurrence. Lines indicate co-occurrence relationships between terms within the same publication. A clustering algorithm divides the nodes into clusters of different colors, representing distinct research sub-themes, including functional genomics (red), Thoroughbred genetics and association analysis (blue), and population diversity (green).

The cumulative frequency plot of high-frequency keywords from 2011 to 2025 (Figure 6A) illustrates the dynamic evolution of research priorities in this field. Terms representing molecular mechanisms and phenotypic traits—particularly “gene expression,” “gene,” and “heritability”—exhibit a sustained and robust upward trend. The continuous growth of “gene expression” signifies that the discipline has moved beyond traditional pedigree-based breeding to explore the transcriptional regulation and molecular mechanisms underlying performance traits. To further elucidate the knowledge structure of this field, a co-occurrence network of keywords was constructed (Figure 6B). This network reveals several distinct thematic clusters. The blue cluster (Genetics and Breeding), centered around nodes such as “thoroughbred horse,” “association,” and “myostatin,” represents genome-wide association studies (GWAS) and the identification of specific genetic variants associated with racehorse performance. The red cluster (physiology and gene expression), containing nodes such as “horse,” “expression,” and “skeletal muscle,” highlights functional genomics research, focusing on how gene transcription levels influence muscle development and physiological responses to exercise. The green cluster (Population Genetics) contains terms related to genetic diversity and phylogenetic analysis, reflecting research on the evolutionary history of different horse breeds and the conservation of genetic resources.

#### 3.3.2. Keyword Emergence Analysis and Emerging Trends

**Figure 7:**
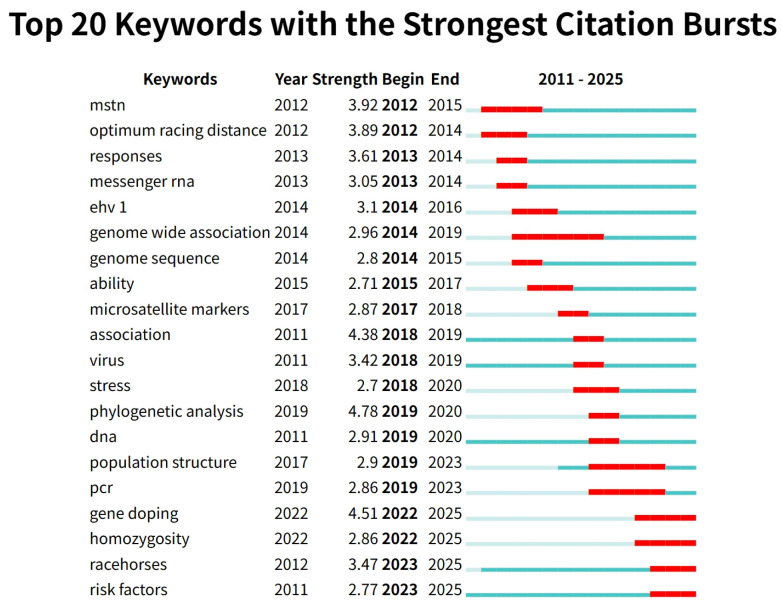
Top 20 keywords with the highest emergence strength in the field of horse racing genetics (2011–2025). “Strength” indicates the magnitude of the sudden surge in the frequency of a keyword over a short period. The blue line represents the entire research period (2011–2025), while the red line represents the specific time span during which the keyword exhibited significant emergence. The light blue lines represent the time periods during which the keywords were present in the published literature but did not show a citation burst.

Word emergence analysis (Figure 7) indicates that current research is focusing on the top 20 keywords with the strongest citation emergence between 2011 and 2025. “Phylogenetic analysis” (emergence strength = 4.78), “gene doping” (emergence strength = 4.51), and “association” (emergence strength = 4.38) exhibit the highest emergence strengths. The keywords “genome-wide association” (2014–2019), “population structure” (2019–2023), and “PCR” (2019–2023) have received the longest-lasting attention over the past period. The keywords “gene doping” (2022–2025), “homozygosity” (2022–2025), “racehorses” (2023–2025), and “risk factors” (2023–2025) have been used more frequently recently. To further summarize representative evidence from these emerging research areas, key studies and their main findings are listed in Table 4. This indicates that these keywords have garnered significant attention recently and are likely to become hot research topics in the future. The molecular limits of manes and their complex physiological response mechanisms.

#### 3.3.3. Most Cited Articles

An analysis of high-impact papers in the field of horse genetics (Supplementary Table S1) reveals a pronounced head effect in citation frequency within the academic discourse of this field. The article by Couëtil LL (2016) [28] published in J Vet Intern Med ranks first with a total of 341 citations. Notably, the study by Li VL (2022) [29] published in Nature demonstrated exceptional academic impact, with both its annual citation rate (46.00) and normalized citation frequency (21.99) far exceeding those of earlier literature. Highly cited papers are primarily concentrated in journals such as *Nature*, *PLoS Genetics*, *Equine Vet J*, and *Scientific Reports.* Research topics encompass equine genetic diversity, stem cell therapy, exercise physiology, and gene regulation related to endurance, constituting the core knowledge base of research in this field.

#### 3.3.4. Key Article Citation Burst Map

**Figure 8:**
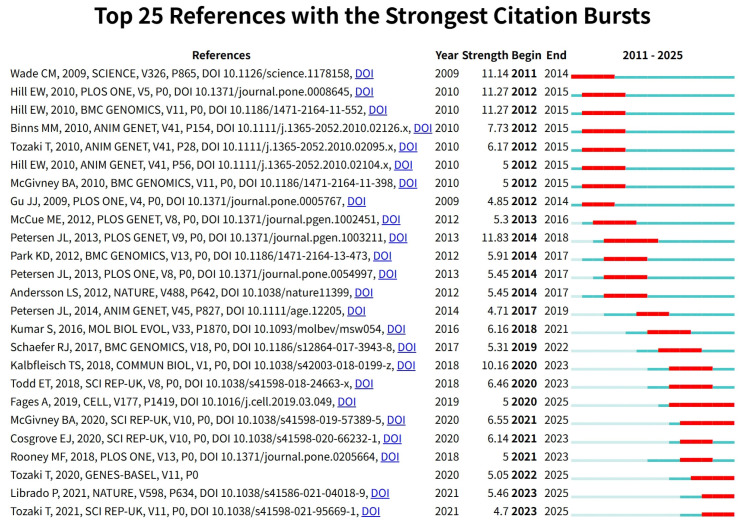
The top 25 most highly cited papers in the field of horse genetics research.

Analysis of the 25 publications with the strongest citation bursts (Figure 8) reveals that research in this field has undergone distinct phased evolution. The early period (2009–2015) was centered on Wade CM’s (2009) [30] genome sequencing and Hill EW’s (2010) [31,32] research on exercise-related genes, which laid the foundation for the discipline. In the middle phase (2014–2018), the study by Petersen JL (2013) [33] on genetic diversity exhibited the highest burst intensity (Strength = 11.83). Recently (2020–present), with contributions from scholars such as Fages A (2019) [34] and Tozaki T (2021) [35] in the fields of ancient genomics and genome-wide association studies, the research frontier has shifted toward complex evolutionary mechanisms and polygenic integration analysis, and several studies remain in a period of sustained growth.

### 3.4. Sankey Diagram

**Figure 9:**
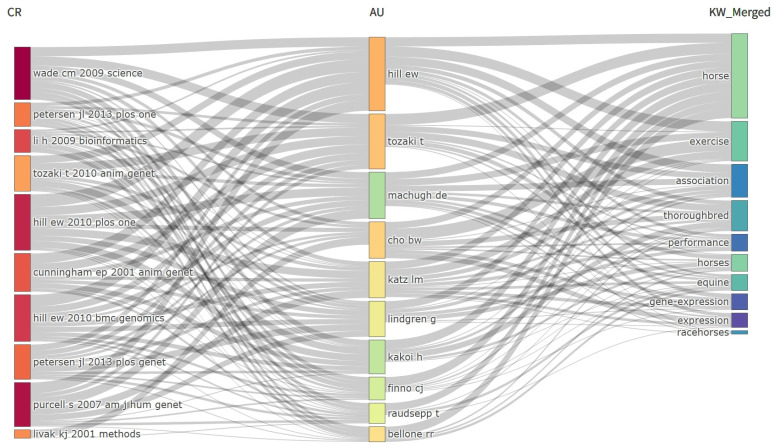
A tri-view diagram showing cited references (**left**), authors (**center**), and keywords (**right**). Node size represents weight, and line thickness represents the strength of the association. The grey lines (flows) indicate the connections between cited references (CR), authors (AU), and merged keywords (KW_Merged), where the thickness of the lines represents the strength of the links.

The Sankey diagram analysis (Figure 9) reveals the close connections between core authors, highly cited papers, and research topics in this field. Core authors such as Hill EW, Tozaki T, and Machugh DE have not only contributed a large number of publications but have also focused research efforts on gene expression and association analyses related to “exercise,” “performance,” and “thoroughbreds” by citing seminal studies such as Wade CM (2009) [30] and Hill EW (2010) [31,32]. The results indicate that research in this field is highly concentrated on the genetic basis of racehorse performance and has formed a mature academic network centered around core experts.

## 4. Discussion

This study indicates that the number of publications on horse genetics showed an overall upward trend from 2011 to 2025, suggesting that the field has gradually transitioned from its initial stages to a phase of stable development. In 2018, the EquCab3.0 reference genome, constructed using advanced technologies such as long-read sequencing, was released. Its improved continuity and completeness provided an important foundation for advances in research quality and productivity after 2019 [36,37]. The sustained publication activity observed since 2022 further supports the continued vitality of this research area [38]. Equine genetics research has progressively transitioned from fragmented exploratory studies to a globally interconnected research system driven by advances in genomic technologies. Leading institutions such as UC Davis and University College Dublin [39], together with influential researchers, have established a stable international research network spanning North America, Europe, and East Asia.

From a global perspective, the United States continues to play a dominant role. Leveraging its long-standing and well-established research infrastructure, the U.S. maintains a leading position in equine genomics, particularly in the construction of equine reference genomes (such as EquCab3.0) [40], the validation of gene variants associated with muscle fiber types (MYH1/MYH2) [41,42], and functional studies of excitability-related polymorphisms (DRD2/SLC6A3) [43]. This is primarily attributable to its long-standing expertise and well-established research infrastructure in the fields of animal genetics and veterinary medicine [44]. However, in recent years, Asian countries, led by China, have seen a rapid increase in publication output, demonstrating a clear late-mover advantage. This trend is not only related to sustained national-level investment in livestock genetic breeding research but also reflects the accelerated adoption of genomic technologies such as high-throughput sequencing [10]. At the same time, growing demand for research on racehorses and related economic animals has further strengthened the applied orientation of this field [45,46]. It is worth noting that although the global research landscape is shifting from a single-polar model toward multipolar collaboration, significant gaps still exist among different countries in terms of research depth, originality, and international influence.

Network analysis of collaborations further reveals that while geographical proximity still influences collaboration patterns to some extent, intercontinental collaboration has become a key driver of progress in this field [38]. Such collaborations have significantly advanced integrated research among horse breeds with different genetic backgrounds—such as comparative analyses of Western Thoroughbreds and Central Asian native breeds—thereby elucidating the molecular mechanisms underlying athletic performance and environmental adaptation within a broader genetic context [38,47,48]. However, collaboration remains concentrated around a limited number of leading institutions, highlighting the need for broader participation and more equitable access to shared genomic resources [49]. A small number of core institutions, such as leading equine genomics centers in the United States and Europe, dominate knowledge production and data resources [50], while the depth of participation by some emerging research forces in the global network remains to be enhanced. The future development of equine genetics research will depend on institutionalized cross-regional collaboration mechanisms, standardized platforms for sharing phenotypic and genotypic data [51], and the establishment of open science infrastructure [52,53] which will be key directions for advancing equine genetics research to a higher level.

Based on keyword emergence analysis, research themes in horse genetics have followed a relatively continuous evolutionary trajectory over the past decade or so, with research focus gradually shifting in tandem with advances in sequencing technology and the expansion of research questions. Early research (approximately 2011–2015) primarily centered on keywords such as MSTN [54,55,56,57,58,59] and messenger RNA [60,61], with most relevant literature focusing on the functional characterization of candidate genes and their mechanisms of action in skeletal muscle development and the formation of motor ability. For example, studies on MSTN generally examined its potential contribution to the regulation of muscle fiber types and differences in athletic performance, while transcriptomic analyses were primarily used to elucidate the relationship between gene expression and exercise adaptation [62]. Research during this phase was generally still based on single genes or limited gene sets, with a focus on establishing preliminary correlations between athletic traits and molecular genetic factors. With the widespread adoption of high-throughput sequencing technologies, genome-wide association studies (GWAS) made significant progress in the field of sports science between 2014 and 2019, providing a systematic approach to elucidating the genetic basis of complex athletic traits [63,64]. Through genome-wide multi-locus scans, these studies conducted systematic association analyses of speed, endurance, and susceptibility to certain sports-related diseases [65,66,67]. For example, regarding endurance traits in racehorses, a 2017 GWAS study aimed to identify the genetic basis for Arabian horses’ adaptation to extreme endurance exercise [66]. Research during this phase gradually expanded from single-gene candidate studies to population-level genetic variation analysis, broadening the understanding of athletic traits from localized mechanisms to the level of the overall genomic architecture [65,66]. In recent years, research foci have further expanded, with keywords such as “gene doping,” “homozygosity,” and “risk factors” increasingly appearing in the literature (see Table 4). In gene doping-related research, the focus is shifting from “target identification” to “precision regulation and systematic validation”. Currently, highly sensitive PCR technologies (such as ddPCR) have effectively resolved the detection bottleneck associated with trace samples [68,69], while multiplex detection platforms (such as πCode) have significantly improved throughput efficiency [70]. A notable shift in research focus is reflected in the extensive use of next-generation sequencing (NGS), which not only enables unbiased identification of structural artificial modifications via whole-genome sequencing (WGS) but also enhances the forensic reliability of evidence through bioinformatics methods [35,71,72]. Concurrently, research on homozygosity has been increasing; the heterogeneous distribution of Runs of Homozygosity (ROH) within the Thoroughbred genome highlights the trade-off between selection for athletic performance and the maintenance of genetic diversity [22,73,74]. Although intense directed selection has resulted in significant genetic fixation at core loci (such as MSTN) related to muscle growth and metabolic regulation, significantly enhancing athletic performance [6], the resulting increase in genome-wide homozygosity may also contribute to inbreeding depression [22]. The accumulation of high-density ROH segments has been associated with shortened athletic lifespan and reduced reproductive fitness in horses, likely due to the increased homozygosity of deleterious recessive alleles across the genome [74]. In light of this, this study argues that horse breeding urgently needs to integrate molecular tools such as genomic kinship matrices to scientifically limit the accumulation of genetic load while pursuing genetic gains [6,75]. Such a balanced strategy may help mitigate the tension between performance improvement and long-term population viability and achieving the sustainable utilization of equine genetic resources [76]. Risk factors and exercise-induced disorders are influenced by interactions between genetic susceptibility and environmental exposure within the Genetic Load (G) × (E) Environmental Stressors framework [77]. While long-term breeding practices have fixed beneficial alleles to enhance performance, they may also increase the frequency of alleles associated with injury susceptibility [6,78]. Among these, variations in structural genes and core metabolic genes may contribute to an underlying susceptibility to injury from the dimensions of physical strength and physiological resilience, respectively, while training load-induced mechanical feedback and biosecurity pressures accelerate the transition to pathological phenotypes [79,80,81,82]. These interacting mechanisms suggest that racehorse health management is gradually shifting from “post-event intervention” to “systematic early warning” [83]. This trend signifies a shift in the field from simple predictions of genetic potential to multidimensional functional assessments that integrate environmental stress and health risks. Equine genetics research is increasingly focused on integrating performance, health, and sustainability [51]. Balancing breeding efficiency with genetic diversity and long-term population health will remain a major challenge for future research in this field [84].

Through a cross-validation of global research landscape (three-field map) and shifts in academic thought (citation emergence), this study found that the United States, Australia, and the United Kingdom are not only key hubs of research output but have also led the transition from ‘single-gene association analysis’ to ‘multidimensional genome-wide systematic analysis’ [85]. Early breakthroughs represented by Hill EW (2010) [31,32] and Wade CM (2009) [30], laid the foundation for molecular breeding, while the continued emergence of recent literature represented by Librado P (2021) [86] and Tozaki T (2021) [35], among others, (continuing through 2025) signifies that the global perspective has evolved from localized performance improvement to a deep integration of “global population evolution” and “precision genomic selection” [87,88]. This trend provides an important theoretical framework for the precision breeding of local horse breeds.

Through the triangulation of Bibliometrix, VOSviewer, and CiteSpace, this study systematically deconstructed the trajectory of knowledge evolution and core logic in equine genetics over the past 15 years. While rigorous data cleaning was performed to ensure the robustness of the analysis, this study has certain limitations; due to its reliance on the Web of Science Core Collection, it may have overlooked some regional technical reports or studies published in non-English journals. Future research could explore integrating data such as patents to more comprehensively evaluate the efficiency of breeding translation and the effectiveness of technical practices in the global horse racing industry.

## 5. Conclusions

Research on horse genetics has continued to develop steadily from 2011 to 2025, giving rise to a global collaborative knowledge network. North America and Europe remain the primary contributors, while East Asia’s influence is rapidly growing. The evolution of research themes indicates a shift from candidate gene discovery to genome-wide association studies and multi-omics integration, with a focus on deeper exploration of complex traits influencing athletic performance. Emerging topics such as “gene doping,” “homozygosity,” and “risk factors” reflect a growing concern for genetic safety and sustainable breeding. Overall, horse genetics is shifting from performance-oriented selection toward a comprehensive framework that integrates performance, health, and genetic diversity, providing important guidance for precision breeding and the long-term sustainable development of the horse population.

## Figures and Tables

**Table 1:** Statistical Analysis of the Top 10 Core Journals by Publication Volume in the Field of Horse Racing Genetics.

Ranking	Sources	Articles	Country	IF	JCR-C
1	Animals	57	Switzerland	3	Q1
2	Equine Veterinary Journal	54	UK	2.4	Q1
3	Journal of Equine Veterinary Science	54	USA	1.3	Q2
4	Animal Genetics	37	UK	2.6	Q1
5	Plos One	32	USA	2.9	Q2
6	Livestock Science	24	The Netherlands	1.7	Q2
7	Genes	21	Switzerland	2.8	Q2
8	Scientific Reports	20	UK	3.8	Q2
9	Veterinary Journal	20	UK	2.4	Q1
10	BMC Veterinary Research	16	UK	2.3	Q1

**Table 2:** The 10 Most Influential Authors in Horse Genetics Research from 2011 to 2025.

Ranking	Author	Articles
1	HILL EW	32
2	TOZAKI T	31
3	CHO BW	27
4	BELLONE RR	20
5	KATZ LM	20
6	LINDGREN G	20
7	RAUDSEPP T	20
8	FINNO CJ	19
9	KAKOI H	19
10	MACHUGH DE	19

**Table 3:** Top 10 Most Productive Institutions in Equine Genetics Research (2011–2025).

Ranking	Affiliation	Articles
1	UNIVERSITY OF CALIFORNIA SYSTEM	111
2	UNIVERSITY OF CALIFORNIA DAVIS	103
3	UNIVERSITY COLLEGE DUBLIN	81
4	PUSAN NATIONAL UNIVERSITY	55
5	SEOUL NATIONAL UNIVERSITY (SNU)	54
6	UNIVERSITY OF KENTUCKY	46
7	TEXAS AANDM UNIVERSITY COLLEGE STATION	45
8	TEXAS AANDM UNIVERSITY SYSTEM	45
9	UNIVERSITY OF LONDON	45
10	SWEDISH UNIVERSITY OF AGRICULTURAL SCIENCES	39

**Table 4:** Scientific foundations of the three emerging frontiers in racehorse genetics.

Frontier	Core Scientific Questions	Representative Findings & Mechanisms	Key References (from the 869 Papers)
Gene Doping	Detection of artificial genetic modification and anti-doping surveillance	Development of targeted resequencing, long-read sequencing, and qPCR approaches for identifying exogenous transgenes and gene-editing signatures (e.g., MSTN).	Tozaki et al., 2022 [19] Jiang et al., 2022 [20] Maniego et al., 2023 [21]
Homozygosity	Genomic diversity, inbreeding, and selective breeding effects	ROH and SNP-based analyses reveal associations between homozygosity, inbreeding depression, inherited disorders, and selection signatures linked to athletic traits.	Hill et al., 2022 [22] Esdaile et al., 2022 [23] Gmel et al., 2024 [24]
Risk Factors	Genetic and environmental determinants of health and performance	GWAS and transcriptomic studies identify susceptibility loci for injury and exercise intolerance, while epidemiological studies highlight disease and management-related risks.	Corbin et al., 2012 [25] Norton et al., 2016 [26] Holtby et al., 2023 [27]

## Data Availability

The data presented in this study are available upon request from the corresponding author.

## Supplementary Materials

The following supporting information can be downloaded at: https://www.mdpi.com/article/10.3390/ani16111705/s1, Table S1. Most Global Cited Top 50.
